# Systemic Lupus Erythematosus and Risk of Dry Eye Disease and Corneal Surface Damage: A Population-Based Cohort Study

**DOI:** 10.3390/ijerph20053776

**Published:** 2023-02-21

**Authors:** Ching-Han Tseng, Ying-Hsuan Tai, Chien-Tai Hong, Ying-Xiu Dai, Tzeng-Ji Chen, Yih-Giun Cherng, Shih-Chung Lai

**Affiliations:** 1 Department of Ophthalmology, Shuang Ho Hospital, Taipei Medical University, New Taipei City 23561, Taiwan; 2 Department of Ophthalmology, School of Medicine, College of Medicine, Taipei Medical University, Taipei 11031, Taiwan; 3 Department of Anesthesiology, Shuang Ho Hospital, Taipei Medical University, New Taipei City 23561, Taiwan; 4 Department of Anesthesiology, School of Medicine, College of Medicine, Taipei Medical University, Taipei 11031, Taiwan; 5 Department of Neurology, Shuang Ho Hospital, Taipei Medical University, New Taipei City 23561, Taiwan; 6 Department of Neurology, School of Medicine, College of Medicine, Taipei Medical University, Taipei 11031, Taiwan; 7 Department of Dermatology, Taipei Veterans General Hospital, Taipei 11217, Taiwan; 8 School of Medicine, National Yang Ming Chiao Tung University, Taipei 11221, Taiwan; 9 Department of Family Medicine, Taipei Veterans General Hospital, Taipei 11217, Taiwan; 10 Department of Family Medicine, Taipei Veterans General Hospital, Hsinchu Branch, Hsinchu 31064, Taiwan

**Keywords:** complication, corneal erosion, keratoconjunctivitis sicca, ocular manifestation, peripheral ulcerative keratitis

## Abstract

Systemic lupus erythematosus (SLE) potentially involves multiple parts of the ocular system, including the lacrimal glands and the cornea. The present study sought to assess the risk of aqueous-deficient dry eye disease (DED) and corneal surface damage in patients with SLE. We conducted a population-based cohort study using Taiwan’s National Health Insurance research database to compare the risks of DED and corneal surface damage between subjects with and without SLE. Proportional hazard regression analyses were used to calculate the adjusted hazard ratio (aHR) and 95% confidence interval (CI) for the study outcomes. The propensity score matching procedure generated 5083 matched pairs with 78,817 person-years of follow-up for analyses. The incidence of DED was 31.90 and 7.66 per 1000 person-years in patients with and without SLE, respectively. After adjusting for covariates, SLE was significantly associated with DED (aHR: 3.30, 95% CI: 2.88–3.78, *p* < 0.0001) and secondary Sjögren’s syndrome (aHR: 9.03, 95% CI: 6.86–11.88, *p* < 0.0001). Subgroup analyses demonstrated that the increased risk of DED was augmented among patients with age < 65 years and female sex. In addition, patients with SLE had a higher risk of corneal surface damage (aHR: 1.81, 95% CI: 1.35–2.41, *p* < 0.0001) compared to control subjects, including recurrent corneal erosion (aHR: 2.98, 95% CI: 1.63–5.46, *p* = 0.0004) and corneal scar (aHR: 2.23, 95% CI: 1.08–4.61, *p* = 0.0302). In this 12-year nationwide cohort study, we found that SLE was associated with increased risks of DED and corneal surface damage. Regular ophthalmology surveillance should be considered to prevent sight-threatening sequelae among patients with SLE.

## 1. Introduction

Dry eye disease (DED) is a multifactorial disorder that is characterized by the disruption of tear film homeostasis [1]. Tear film instability and hyperosmolarity, ocular surface inflammation and damage, and neurosensory abnormalities play etiological roles in developing ocular symptoms, including punctate epithelial keratitis, filamentary keratitis, superior limbic keratoconjunctivitis, lid parallel conjunctival folds, and lid wiper epitheliopathy [2]. The prevalence of DED varies globally, ranging from 5 to 50% across different countries and regions [3]. In Taiwan, the prevalence rate of DED was reported to be 5% to 34%, with females and the elderly in the majority [4,5,6,7]. It should be noted that patients with DED have a significantly higher risk of corneal surface damage due to progressive ocular surface inflammation and disruption [3]. Recurrent corneal erosion, corneal ulcers, and corneal scars represent common findings among patients with severe corneal surface damage. Previous studies have revealed several risk factors for DED-associated corneal surface damage, including younger age, female sex, diabetes mellitus, and autoimmune diseases (e.g., rheumatoid arthritis) [8]. Importantly, DED symptoms have an adverse impact on patients’ visual functions, daily activities, work productivity, and vision-related quality of life [9].

Systemic lupus erythematosus (SLE) is a chronic, complex, and multifaceted autoimmune disorder, while its etiology remains largely unclear [10]. SLE predominantly affects females, especially in their 20 s and 30 s [10]. The prevalence of SLE varies across different countries [11]. In Taiwan, the prevalence rate was reported to be 97.5 per 100,000 population [12]. Approximately one-third of SLE patients suffer from ocular involvement, of which keratoconjunctivitis sicca represents the most common manifestation [13,14,15,16]. In a previous report, the risks of DED, cataracts, and glaucoma were significantly higher in patients with SLE [17]. However, there is limited population-based data demonstrating the association between SLE and DED or serious corneal surface damage. The relationship between SLE and DED is not completely clarified due to some methodological drawbacks of previous studies, including small sample size (*n* < 1000) [14,16,17], insufficient adjustment for confounders [14,16,17], and restriction to single institutions [14,16] or specific populations (children) [14]. In addition, few studies have evaluated the potential impact of SLE on the development of corneal surface damage, and relevant risk factors remain largely unknown [14,16,17]. In this population-based cohort study, we used Taiwan’s National Health Insurance (NHI) research database to evaluate the temporal relationship between SLE and DED or corneal surface damage. Based on the current literature [13,14,15,16,17], we hypothesized that SLE was associated with both DED and corneal surface damage in this 12-year nationwide cohort.

## 2. Material and Methods

### 2.1. Data Source 

This study obtained ethical approval from the Taipei Medical University-Joint Institutional Review Board (approval no. TMU-JIRB-N202210011; date of approval on 6 October 2022). Written informed consent was waived by the Institutional Review Board due to the retrospective nature of this research. All methods were performed following the Declaration of Helsinki 2013 and relevant study guidelines [18]. Taiwan’s National Health Insurance program was launched in March 1995 and offered insurance to more than 99% of 23.3 million Taiwanese residents. The NHI research database contains comprehensive claims data of the insured beneficiaries, including demographic characteristics (e.g., date of birth and sex), medical diagnoses, prescription drugs, and medical expenditures. The NHI research database has been widely used for public health statistics and risk assessment [19,20,21]. In the present study, we included subjects from the three Longitudinal Health Insurance Databases (LHID2000, LHID2005, and LHID2010), which contains original claims data of 1 million randomly sampled beneficiaries from the original NHI research database in the years 2000, 2005, and 2010, respectively [22].

### 2.2. Inclusion and Exclusion Criteria

Patients who had at least 2 rheumatology clinic visits with the diagnoses of SLE between 1 January 2002 and 30 June 2013 were included consecutively. We utilized the International Classification of Diseases, 9th Revision, Clinical Modification (ICD-9-CM) codes to ascertain the diagnoses of SLE, coexisting diseases, and ocular disorders (Supplementary Table S1). The index date was defined as the date of the first SLE diagnosis. Patients were excluded due to the following conditions: any previous diagnoses of DED, corneal ulcers, recurrent corneal erosion, corneal scars, interstitial and deep keratitis, corneal neovascularization, ocular burns, or open globe injury in the ophthalmology service before the index date. Subjects were also excluded if they had been prescribed eye lubricants before the index date or died in the follow-up period. 

### 2.3. Outcome Assessment

The primary outcome was DED, which was defined as the diagnosis made twice by certified ophthalmologists with the prescriptions of cyclosporine ophthalmic emulsion in the ophthalmology care service (Supplementary Table S1). In the reimbursement regulations of Taiwan’s National Health Insurance, cyclosporine ophthalmic emulsion treatment can be used when patients’ Schirmer test scores are less than 5 mm in 5 min [5,8]. The secondary outcomes included secondary Sjögren’s syndrome (SS) and severe forms of corneal surface damage, which were defined as any diagnosis of corneal ulcers, recurrent corneal erosion, or corneal scars made twice by certified ophthalmologists.

### 2.4. Covariates for Model Adjustment

Insurance premium was classified into $0–$500, $501–$800, and >$800 United States dollars per month. The ICD-9-CM codes of physicians’ diagnoses within 24 months before the index date were employed to determine the following comorbidities, chosen based on data availability and existing literature: hypertension, diabetes mellitus, coronary artery disease, chronic obstructive pulmonary disease, chronic liver disease, chronic kidney disease, cerebrovascular disease, thyroid disease, major depressive disorder, anxiety disorder, sleeping disorder, and cancer (Supplementary Table S1) [23]. The Charlson comorbidity index score was calculated to evaluate the comorbidity level of included subjects [24]. We also evaluated the concurrent prescription of systemic corticosteroids within 6 months after the index date. The numbers of hospitalizations and emergency visits within 24 months before the index date were analyzed to assess the level of medical resource utilization of the studied patients.

### 2.5. Statistical Analysis

A non-parsimonious multivariable logistic regression model was used to calculate a propensity score for SLE and non-SLE subjects. Each SLE subject was matched to a non-SLE control using the nearest neighbor matching algorithm within a tolerance limit of 0.05 and without replacement to balance the distributions of age, sex, and monthly insurance premium between the two groups [25]. Baseline patient characteristics were compared between matched pairs using the absolute standardized mean difference [26]. We used multivariable Cox proportional hazards regression models to calculate the adjusted hazard ratio (aHR) and 95% confidence interval (CI) for the study outcomes. The multivariable models adjusted for the variables of age, sex, monthly insurance premium, coexisting diseases, Charlson comorbidity index score, use of systemic corticosteroids, number of hospitalizations, and number of emergency room visits. The Kaplan-Meier curves and log-rank tests were used to compare the cumulative incidence of ophthalmological outcomes between the two groups. Stratified analyses were also conducted by age≥ or <65 years, male or female, different Charlson comorbidity index scores, and use of systemic corticosteroids or not to examine the risk of DED within these strata. A two-sided *p*-value of <0.05 was considered statistically significant. All the statistical analyses were conducted using Statistics Analysis System (SAS), Version 9.4 (SAS Institute Inc., Cary, NC, USA). 

## 3. Results

### 3.1. Baseline Patient Characteristics

The matching procedure generated 5083 matched pairs with 78,817 person-years of follow-up for analyses (Supplementary Figure S1). The baseline distributions of demographic and patient characteristics are shown in Table 1. Noticeably, patients with SLE were more likely to have more comorbidities, higher Charlson comorbidity index scores, prescriptions of systemic corticosteroids, and greater numbers of hospitalizations and emergency room visits.

### 3.2. Dry Eye Disease

**Figure 1:**
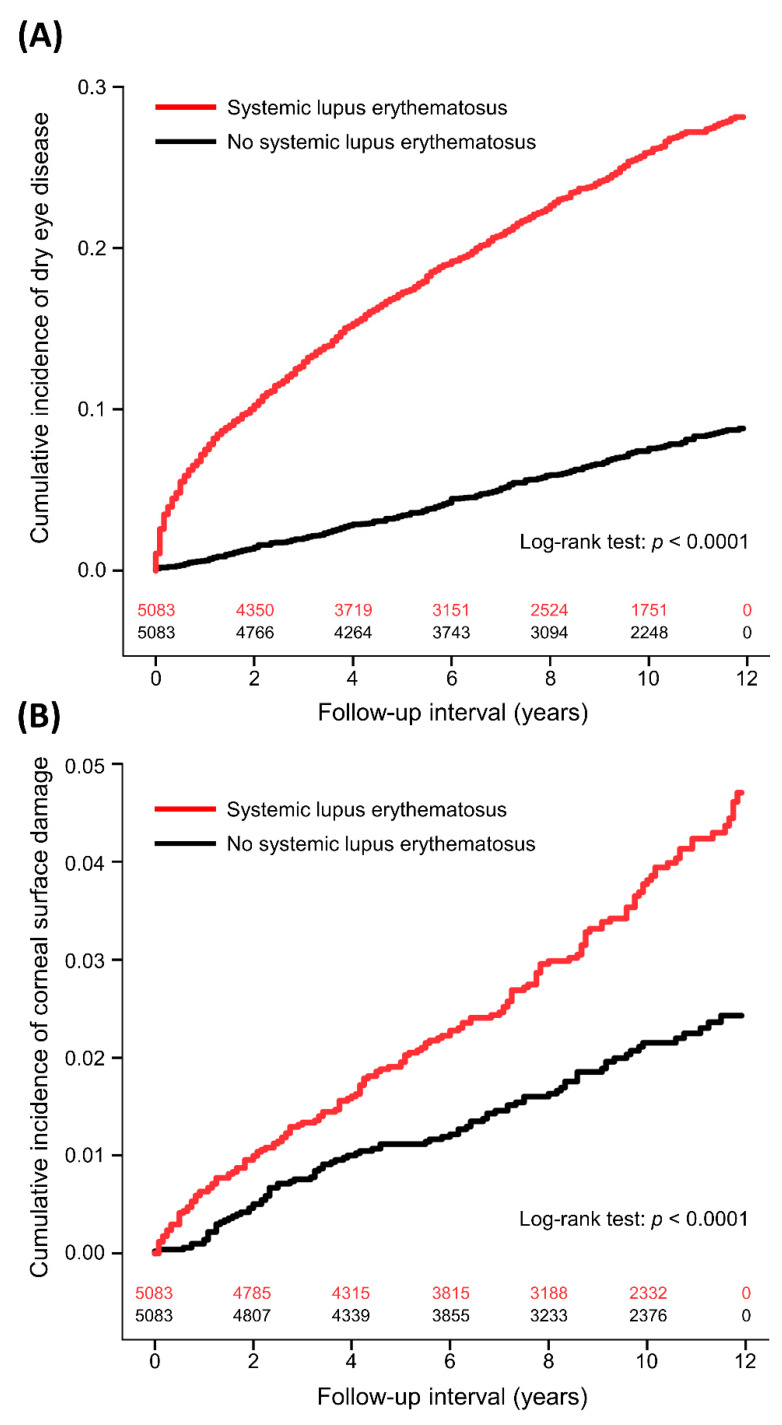
Cumulative incidence of dry eye disease (**A**) and corneal surface damage (**B**) between patients with and without systemic lupus erythematosus with number of subjects at risk.

The incidence of DED was 31.90 and 7.66 per 1000 person-years in the SLE and non-SLE groups, respectively (Table 2). The interval between enrollment and DED diagnosis was median 2.6 (interquartile range: 0.6–5.6) years in the SLE patients and 5.1 (2.3–7.5) years in the non-SLE controls (*p* < 0.0001). The results of univariate and multivariable proportional hazards regression analyses for DED were shown in Table 3. After adjusting for covariates, SLE was significantly associated with increased DED compared to non-SLE controls (aHR: 3.30, 95% CI: 2.88–3.78, *p* < 0.0001). Figure 1A demonstrates the cumulative incidence of DED in the two groups. SLE was also linked to secondary SS (aHR: 9.03, 95% CI: 6.86–11.88, *p* < 0.0001). Other independent factors for DED were age (aHR: 1.03), female sex (aHR: 2.56), monthly insurance premium (501–800 vs. 0–500 USD, aHR: 0.92; ≥801 vs. 0–500 USD, aHR: 1.20), hypertension (aHR: 0.78), cerebrovascular disease (aHR: 0.68), sleeping disorder (aHR: 1.24), Charlson comorbidity index (1 vs. 0, aHR: 1.45; 2 vs. 0, aHR: 1.35; ≥3 vs. 0, aHR: 0.74), and use of systemic corticosteroids (aHR: 1.41). Subgroup analyses showed that the aHR for DED was higher in patients with age < 65 years (aHR: 3.48) and female sex (aHR: 3.47) compared to those with age ≥ 65 years (aHR: 1.99) and male sex (aHR: 2.20), respectively (Table 4). 

### 3.3. Corneal Surface Damage

The incidence of corneal surface damage was 3.93 and 2.12 per 1000 person-years in the SLE and non-SLE groups, respectively (Table 2). The time to corneal surface damage was median 4.3 years (interquartile range: 1.7–7.8) in the SLE patients and 3.8 years (interquartile range: 2.0–7.4) in the non-SLE controls (*p* = 0.9610). The multivariable model showed that SLE was significantly associated with increased corneal surface damage (aHR: 1.81, 95% CI: 1.35–2.41, *p* < 0.0001; Table 5 and Figure 1B). Further analyses showed that SLE was significantly associated with higher risks of recurrent corneal erosion (aHR: 2.98, 95% CI: 1.63–5.46, *p* = 0.0004) and corneal scar (aHR: 2.23, 95% CI: 1.08–4.61, *p* = 0.0302). Another independent factor for corneal surface damage was female sex (aHR: 1.94, 95% CI: 1.28–2.94, *p* = 0.0017).

## 4. Discussion

The present study demonstrated that patients with SLE exhibited significantly greater risks of DED and corneal surface damage, especially for recurrent corneal erosion compared with age, sex and insurance premium-matched controls. Subgroup analyses further revealed that the higher SLE-associated risk of DED was observed in subjects of males and females, age ≥ 65 and <65 years old, and those with or without systemic corticosteroid treatment. Considering the devastating impact of DED and corneal surface damage on visual functions, patients with SLE should be alerted on these corneal disorders.

SLE is the third most common autoimmune disorder in Taiwan [27]. The prevalence rate of SLE remarkably increased during the 21st century [28]. Although the ocular symptoms are not included into the 11 diagnostic criteria of SLE, they are not uncommon and about one-third of patients are suffered [29]. Keratoconjunctivitis sicca is the most ocular manifestation of SLE [30], while all the parts of eye, including sclera, uvea, retina, and optic nerve, are possibly involved [31]. There are several reasons accounting for the association between SLE and DED. One is the comorbidity of Sjögren’s syndrome, which causes the reduction of tear. On the other hand, the infiltration of immune cells and immune complex into the epithelial basement membrane are also evident [32], and the increase in proinflammatory cytokine, i.e., interleukin-17, is detected in the tear film of SLE patients [33,34]. The present study also found a significant association between SLE with secondary SS in Taiwanese patients. However, the adjusted risk of DED was similar between SLE patients with and without systemic corticosteroid compared with controls, which may indicate the consequence of an ocular-specific inflammatory response in SLE patients, and topical immunosuppressants are more suitable for the management of DED [35,36].

Corneal ulcer is defined as the lesion of the corneal epithelium, which is a major threat of vision [37]. Without the proper treatment, patients may only rely on the corneal transplant for regaining their vision [38]. Most of the corneal ulcers result from the infection, including bacteria, virus, fungus, and protozoa [38]. On the other hand, non-infectious corneal ulcers, usually present as peripheral ulcerative keratitis (PUK), are highly associated with autoimmune diseases [39]. The fibrocyte and macrophage infiltration in the corneal matrix triggers the inflammatory response, and the accumulation of immune complex is found in the capillary network of cornea in PUK [40,41]. In the present study, the overall risk of corneal surface damage was significantly higher in patients with SLE. Despite that there was only a trend toward increased corneal ulcers in SLE patients, referring to recurrent corneal erosion and corneal scar, more severe types of corneal damage, there were significantly increased risks in SLE patients. The lack of significance in the corneal ulcer among SLE patients may result from other types of PUK, such as infectious or contact lens-related keratitis.

The strength of the present study was the delineation of the association between SLE and DED or corneal surface damage. Meanwhile, the population-based study provided reliable epidemiological evidence and good generalizability about the risk assessment. DED causes substantial discomfort for the SLE patients, and the management of these ocular manifestations of SLE should be emphasized. In addition, despite that SLE is not the major contributor of PUK, it did increase the risk of recurrent corneal ulcer and corneal scar, which may exert a devastating effect on eyesight. However, there were some limitations to the present study. First, since the NHI research database was diagnosis and treatment-based, the laboratory data was unavailable. Therefore, the severity and progression of SLE, DED and corneal surface damage could not be further evaluated. Second, the lack of some social habit information (e.g., alcohol and tobacco consumption) and physical examination data (e.g., body mass index, blood pressure, and visual acuity) might also introduce a bias to the analytical results. Third, we only matched the variables of age, sex and monthly insurance premium between the two groups in the propensity-score matching process in order to increase the sample size and statistical power of matched datasets. Given the fact that the incidence of corneal surface injury was relatively low (approximately 2% to 5% in the 12-year follow-up), a large patient sample is essential to detect a potential risk difference between SLE and non-SLE populations. Fourth, because the use of corticosteroids is a known risk factor for DED and corneal surface damage, the imbalance in the distribution of corticosteroid prescriptions might bias the study results. Further studies are needed to evaluate the potential impact of corticosteroids and immunosuppressants on corneal diseases among SLE patients. Finally, our cohort was only followed up until the 31 December 2013, due to the regulations of the NHI research database. 

## 5. Conclusions

The present study demonstrated a higher risk of DED and severe forms of corneal surface damage in patients with SLE. Considering the increasing prevalence of SLE, the vision issues, which affect the quality of life substantially, should be empathized with the rheumatologists and ophthalmologists. Prophylactic and therapeutic management should be further developed for this susceptible population.

## Figures and Tables

**Table 1:** Baseline characteristics of subjects with and without systemic lupus erythematosus.

	SLE *n* = 5083		Non-SLE *n* = 5083		ASMD
Age (years), mean (SD)	36.1	16.1	36.1	16.1	<0.0001
Sex, female, *n* (%)	4161	81.9	4161	81.9	<0.0001
Insurance premium (USD/month), *n* (%)					<0.0001
0–500	1921	37.8	1921	37.8	
501–800	1561	30.7	1561	30.7	
≥801	1601	31.5	1601	31.5	
Coexisting diseases, *n* (%)					
Hypertension	527	10.4	337	6.6	0.2690
Diabetes mellitus	239	4.7	175	3.4	0.1791
Coronary artery disease	200	3.9	107	2.1	0.3553
Chronic obstructive pulmonary disease	189	3.7	100	2.0	0.3609
Chronic liver disease	383	7.5	172	3.4	0.4656
Chronic kidney disease	98	1.9	21	0.4	0.8577
Cerebrovascular disease	133	2.6	63	1.2	0.4197
Thyroid disease	172	3.4	40	0.8	0.8188
Major depressive disorder	55	1.1	23	0.5	0.4842
Anxiety disorder	501	9.9	243	4.8	0.4291
Sleeping disorder	456	9.0	232	4.6	0.3986
Cancer	109	2.1	55	1.1	0.3831
Charlson comorbidity index					0.6027
0	3435	67.58	4741	93.27	
1	1248	24.55	246	4.84	
2	386	7.59	74	1.46	
≥3	14	0.28	22	0.43	
Use of systemic corticosteroids, *n* (%)	2680	52.72	585	11.51	1.1847
Number of hospitalizations, *n* (%)					0.2569
0	4307	84.73	4713	92.72	
1	545	10.72	295	5.80	
2	132	2.60	49	0.96	
≥3	99	1.95	26	0.51	
Number of emergency room visits, *n* (%)					0.3028
0	3709	72.97	4309	84.77	
1	809	15.92	534	10.51	
2	320	6.30	154	3.03	
≥3	245	4.82	86	1.69	

Abbreviation: ASMD = absolute standardized mean difference; SD = standard deviation; SLE = systemic lupus erythematosus; USD = United States Dollar.

**Table 2:** Risks of dry eye disease, Sjögren’s syndrome, and corneal surface damage for patients with and without systemic lupus erythematosus.

	SLE *n* = 5083		Non-SLE *n* = 5083		Outcome Risk		
Study Outcome	Incident Case	Incidence per 1000 Person-Years	Incident Case	Incidence per 1000 Person-Years	IRR	aHR (95% CI) ^†^	*p*
Dry eye disease	1165	31.90	324	7.66	4.16	3.30 (2.88–3.78)	<0.0001
Sjögren’s syndrome	693	17.54	61	1.40	12.53	9.03 (6.86–11.88)	<0.0001
Corneal surface damage	169	3.93	92	2.12	1.85	1.81 (1.35–2.41)	<0.0001
Corneal ulcer	98	2.28	62	1.43	1.59	1.36 (0.94–1.98)	0.1061
Recurrent corneal erosion	48	1.12	17	0.39	2.87	2.98 (1.63–5.46)	0.0004
Corneal scar	24	0.56	14	0.32	1.75	2.23 (1.08–4.61)	0.0302

Abbreviation: aHR = adjusted hazard ratio; CI = confidence interval; IRR = incidence rate ratio; SLE = systemic lupus erythematosus. †: Adjusted for age (continuous), sex, insurance premium (categorical), coexisting diseases, Charlson comorbidity index score, use of systemic corticosteroids, number of hospitalizations, and number of emergency room visits.

**Table 3:** Univariate and multivariable analyses for dry eye disease.

	Univariate			Multivariable		
	cHR	95% CI	*p*	aHR	95% CI	*p*
Systemic lupus erythematosus	4.06	3.59–4.60	<0.0001	3.30	2.88–3.78	<0.0001
Age (years)	1.02	1.02–1.02	<0.0001	1.03	1.02–1.03	<0.0001
Sex, female vs. male	2.28	1.91–2.72	<0.0001	2.56	2.14–3.06	<0.0001
Insurance premium (USD/month)			0.0583			0.0004
501–800 vs. 0–500	0.86	0.76–0.98	0.0181	0.92	0.81–1.05	0.2162
≥801 vs. 0–500	0.95	0.85–1.08	0.4483	1.20	1.06–1.36	0.0053
Coexisting diseases						
Hypertension	1.42	1.20–1.68	<0.0001	0.78	0.64–0.95	0.0131
Diabetes mellitus	1.30	1.01–1.66	0.0398	0.80	0.61–1.04	0.1003
Coronary artery disease	1.92	1.51–2.45	<0.0001	1.21	0.93–1.57	0.1673
COPD	1.32	0.99–1.75	0.0554	0.89	0.67–1.19	0.4422
Chronic liver disease	1.56	1.29–1.89	<0.0001	1.07	0.87–1.31	0.5078
Chronic kidney disease	1.50	1.00–2.25	0.0485	0.76	0.50–1.16	0.2055
Cerebrovascular disease	1.28	0.90–1.83	0.1694	0.68	0.47–0.99	0.0436
Thyroid disease	2.11	1.60–2.78	<0.0001	1.27	0.96–1.69	0.0944
Major depressive disorder	1.60	0.96–2.67	0.0688	0.99	0.59–1.67	0.9637
Anxiety disorder	1.73	1.46–2.05	<0.0001	1.16	0.96–1.40	0.1154
Sleeping disorder	1.76	1.47–2.10	<0.0001	1.24	1.02–1.50	0.0314
Cancer	1.66	1.16–2.37	0.0053	1.13	0.79–1.62	0.5159
Charlson comorbidity index			<0.0001			<0.0001
1 vs. 0	2.53	2.26–2.84	<0.0001	1.45	1.28–1.64	<0.0001
2 vs. 0	2.20	1.82–2.66	<0.0001	1.35	1.10–1.64	0.0038
≥3 vs. 0	0.97	0.36–2.58	0.9428	0.74	0.28–1.99	0.5506
Use of systemic corticosteroids	2.40	2.17–2.66	<0.0001	1.41	1.25–1.58	<0.0001
Number of hospitalizations			0.5954			0.3859
1 vs. 0	1.10	0.91–1.33	0.3201	0.91	0.74–1.11	0.3432
2 vs. 0	1.20	0.81–1.76	0.3670	0.81	0.54–1.22	0.3156
≥3 vs. 0	1.12	0.68–1.83	0.6577	0.69	0.41–1.19	0.1811
Number of emergency room visits			0.0203			0.6522
1 vs. 0	1.17	1.00–1.36	0.0469	1.03	0.88–1.20	0.7448
2 vs. 0	1.11	0.86–1.43	0.4246	0.88	0.67–1.14	0.3161
≥3 vs. 0	1.43	1.08–1.89	0.0119	1.09	0.80–1.48	0.5893

Abbreviation: aHR = adjusted hazard ratio; COPD = chronic obstruction pulmonary disease; cHR = crude hazard ratio; USD = United States Dollar.

**Table 4:** Subgroup analyses of dry eye disease for patients with and without systemic lupus erythematosus.

	SLE *n* = 5083		Non-SLE *n* = 5083		Outcome Risk		
Subgroup	Incident Case	Incidence per 1000 Person-Years	Incident Case	Incidence per 1000 Person-Years	IRR	aHR (95% CI) ^†^	*p*
All patients	1165	31.90	324	7.66	4.16	3.30 (2.88–3.78)	<0.0001
Age ≥ 65 years	73	40.25	36	17.26	2.33	1.99 (1.30–3.06)	0.0016
Age < 65 years	1092	31.47	288	7.16	4.40	3.48 (3.02–4.02)	<0.0001
Male	97	13.81	37	4.96	2.78	2.20 (1.46–3.31)	0.0002
Female	1068	36.21	287	8.24	4.39	3.47 (3.00–4.00)	<0.0001
CCI score = 0	662	26.80	291	7.41	3.62	3.20 (2.76–3.70)	<0.0001
CCI score = 1	394	44.98	20	9.21	4.88	4.29 (2.67–6.91)	<0.0001
CCI score = 2	106	35.82	12	17.84	2.01	2.45 (1.26–4.77)	0.0085
CCI score ≥ 3	3	29.93	1	5.68	5.27	8.12 (0.03–2021.97)	0.4568
Use of systemic corticosteroids	707	38.30	51	10.09	3.80	3.45 (2.58–4.62)	<0.0001
No use of systemic corticosteroids	458	25.36	273	7.33	3.46	3.27 (2.80–3.83)	<0.0001

Abbreviation: aHR = adjusted hazard ratio; CCI = Charlson comorbidity index; CI = confidence interval; IRR = incidence rate ratio; SLE = systemic lupus erythematosus. †: Adjusted for age (continuous), sex, insurance premium (categorical), coexisting diseases, Charlson comorbidity index score, use of systemic corticosteroids, number of hospitalizations, and number of emergency room visits.

**Table 5:** Univariate and multivariable analyses for corneal surface damage.

	Univariate			Multivariable		
	cHR	95% CI	*p*	aHR	95% CI	*p*
Systemic lupus erythematosus	1.85	1.44–2.39	<0.0001	1.81	1.35–2.41	<0.0001
Age (years)	1.00	0.99–1.01	0.8792	1.00	0.99–1.01	0.8177
Sex, female vs. male	2.00	1.33–3.03	0.0010	1.94	1.28–2.94	0.0017
Insurance premium (USD/month)			0.0729			0.0603
501–800 vs. 0–500	0.88	0.66–1.17	0.3719	0.84	0.63–1.13	0.2521
≥801 vs. 0–500	0.70	0.52–0.95	0.0221	0.69	0.51–0.94	0.0181
Coexisting diseases						
Hypertension	0.86	0.52–1.42	0.5514	0.95	0.54–1.67	0.8461
Diabetes mellitus	0.45	0.17–1.20	0.1100	0.46	0.16–1.27	0.1334
Coronary artery disease	1.07	0.50–2.26	0.8634	1.18	0.53–2.63	0.6919
COPD	0.75	0.31–1.81	0.5217	0.76	0.31–1.88	0.5540
Chronic liver disease	0.86	0.47–1.58	0.6315	0.84	0.45–1.57	0.5835
Chronic kidney disease	0.34	0.05–2.45	0.2863	0.30	0.04–2.19	0.2355
Cerebrovascular disease	0.47	0.12–1.90	0.2893	0.47	0.11–1.94	0.2947
Thyroid disease	0.85	0.32–2.27	0.7389	0.74	0.27–2.02	0.5601
Major depressive disorder	0.59	0.08–4.18	0.5937	0.51	0.07–3.74	0.5073
Anxiety disorder	1.26	0.80–1.99	0.3239	1.16	0.71–1.92	0.5504
Sleeping disorder	1.26	0.77–2.07	0.3539	1.17	0.69–2.00	0.5555
Cancer	0.89	0.29–2.79	0.8466	0.94	0.30–2.99	0.9187
Charlson Comorbidity Index			0.0338			0.3330
1 vs. 0	1.51	1.12–2.03	0.0070	1.22	0.87–1.70	0.2451
2 vs. 0	1.07	0.61–1.88	0.8100	0.92	0.51–1.66	0.7833
≥3 vs. 0	2.54	0.63–10.22	0.1903	2.69	0.66–10.95	0.1670
Use of systemic corticosteroids	1.39	1.08–1.78	0.0099	1.02	0.76–1.36	0.8950
Number of hospitalizations			0.7740			0.6070
1 vs. 0	0.88	0.53–1.46	0.6153	0.78	0.46–1.33	0.3650
2 vs. 0	0.81	0.26–2.54	0.7236	0.66	0.20–2.13	0.4873
≥3 vs. 0	0.42	0.06–2.97	0.3819	0.40	0.05–3.04	0.3772
Number of emergency room visits			0.1484			0.1790
1 vs. 0	1.49	1.06–2.09	0.0229	1.48	1.04–2.10	0.0301
2 vs. 0	0.97	0.50–1.90	0.9336	1.00	0.51–2.00	0.9901
≥3 vs. 0	1.20	0.56–2.54	0.6441	1.29	0.58–2.90	0.5316

Abbreviation: aHR = adjusted hazard ratio; COPD = chronic obstruction pulmonary disease; cHR = crude hazard ratio; USD = United States Dollar.

## Data Availability

The data presented in this study are available on request from the corresponding author.

## Supplementary Materials

The following are available online at https://www.mdpi.com/article/10.3390/ijerph20053776/s1, Table S1: ICD-9-CM codes of exposure factors, coexisting diseases, and study outcomes; Figure S1: Flow diagram for patient selection.
