# Supplementary material for: Systemic Lupus Erythematosus and Risk of Dry Eye Disease and Corneal Surface Damage: A Population-Based Cohort Study
Source: Int J Environ Res Public Health. 2023 Feb 21;20(5):3776. doi: 10.3390/ijerph20053776 (PMC10001508; doi:10.3390/ijerph20053776)
Supplement: Supplementary file 1 [file ijerph-20-03776-s001.zip › Supplementary Table S1.pdf]

**Supplementary Table S1.** ICD-9-CM codes of exposure factors, coexisting diseases, and study outcomes

| | |
|---|---|
| **Exposure factor** | |
| Systemic lupus erythematosus | 710.0, 695.4 |
| **Exclusion criteria** | |
| Dry eye disease | 370.33, 372.53, 375.15, 710.2 |
| Corneal ulcer | 370.0 |
| Recurrent corneal erosion | 371.42 |
| Corneal scar | 371.0 |
| Interstitial and deep keratitis | 370.5 |
| Corneal neovascularization | 370.6 |
| Ocular burn | 940 |
| Open globe injury | 871 |
| **Coexisting disease** | |
| Hypertension | 401-405 |
| Diabetes mellitus | 250 |
| Coronary artery disease | 410-414 |
| Chronic obstructive pulmonary disease | 490, 491, 496 |
| Chronic liver disease | 571 |
| Chronic kidney disease | 585 |
| Cerebrovascular disease | 430-438 |
| Thyroid disease | 240-246 |
| Depressive disorder | 296.2, 296.3 |
| Anxiety disorder | 300 |
| Sleeping disorder | 307.41, 307.42, 327.0, 780.50, 780.52, 292.85 |
| Cancer | 140-208, 230-234 |
| **Study outcome** | |
| Dry eye disease | 370.33, 372.53, 375.15, 710.2 |
| Sjögren's syndrome | 710.2 |
| Corneal ulcer | 370.0 |
| Recurrent corneal erosion | 371.42 |
| Corneal scar | 371.0 |
