# Supplementary figures and images for: Systemic Lupus Erythematosus and Risk of Dry Eye Disease and Corneal Surface Damage: A Population-Based Cohort Study
Source: Int J Environ Res Public Health. 2023 Feb 21;20(5):3776. doi: 10.3390/ijerph20053776 (PMC10001508; doi:10.3390/ijerph20053776)

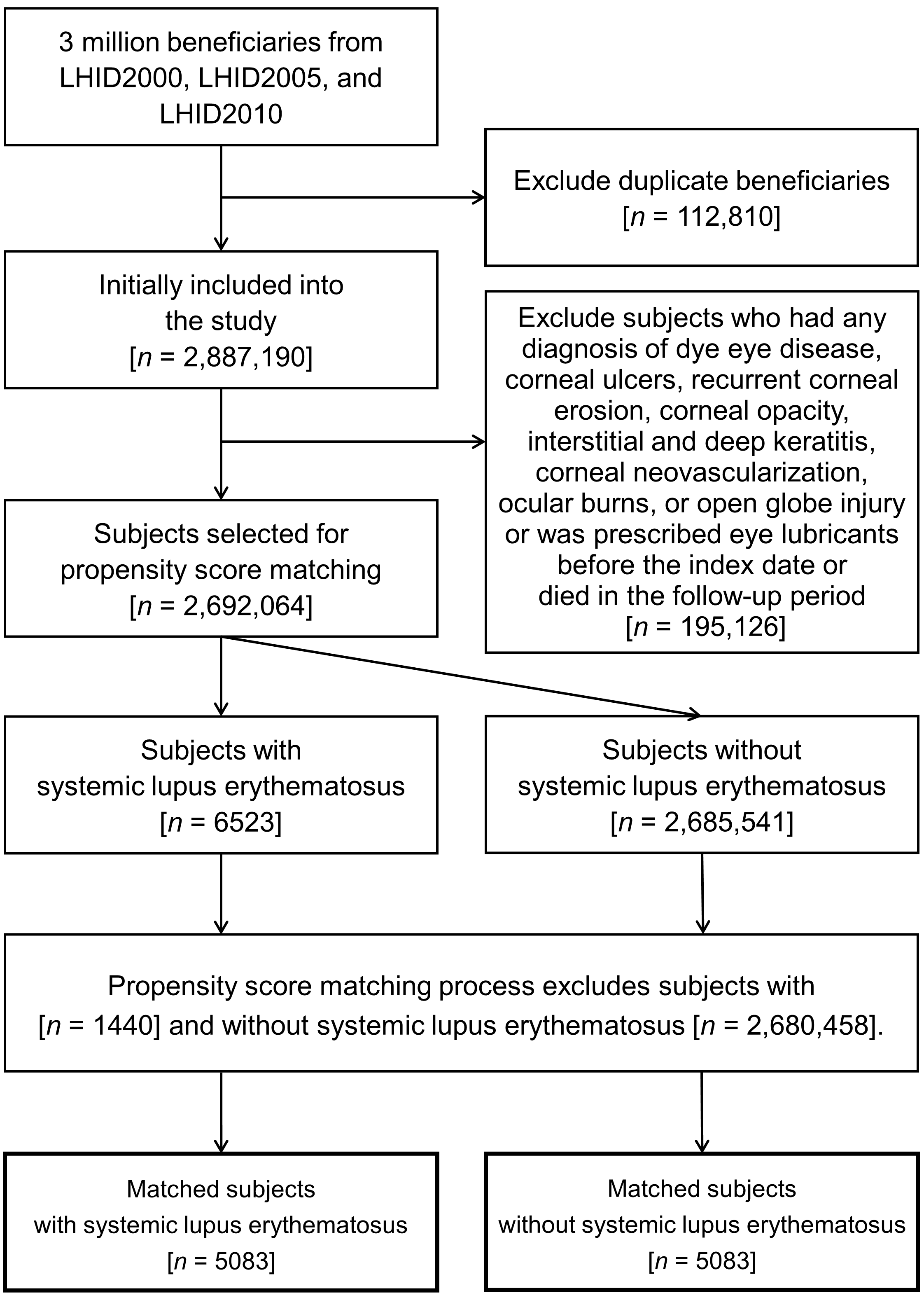

Supplement: Supplementary file 1 [file ijerph-20-03776-s001.zip › Supplementary Figure S1.tif]
